# Women and NCDs: Overcoming the neglect

**DOI:** 10.3402/gha.v7.23742

**Published:** 2014-05-05

**Authors:** Ruth Bonita, Robert Beaglehole

**Affiliations:** University of Auckland, Auckland, New Zealand

**Keywords:** women, gender bias, global health targets, non-communicable diseases, risk factors

## Abstract

Two in every three deaths among women are caused by non-communicable diseases (NCDs) – largely heart disease, stroke, cancer, diabetes and chronic respiratory diseases. The global discourse on health, however, largely views women in terms of their reproductive capacity, a persisting myth reflecting gender bias that shifts the focus away from NCDs, violence, and other injuries. Risk factors for NCDs are similar for men and women. Because fewer women actively smoke than men, and drink in less harmful ways, in most parts of the world, the impact of major NCD risk factors is far less in women than in men. In the area of diagnosis and treatment, gender bias can result in women being asked fewer questions, and receiving fewer examinations and fewer diagnostic tests for coronary heart disease and other NCDs compared with men with similar symptoms. In response to a UN meeting in September 2011, member states of WHO have agreed to a global goal to reduce avoidable NCD mortality by 25% by 2025 (‘25 by 25’). A set of voluntary targets and indictors have been agreed upon, although none of them are gender specific. Most require changes at the policy level that will ensure that women – and children – will also benefit. As the 2015 deadline for the Millennium Development Goals approaches, women and NCDs should be central to the sustainable human development agenda.

Four major transitions are occurring in global health: age, sex, and cause-specific death rates are generally declining; life expectancy is increasing; populations are aging; and there is a dramatic shift from deaths at younger ages, principally due to infectious diseases, to non-communicable diseases (NCDs) – principally cardiovascular diseases (CVDs), cancer, chronic respiratory diseases, and diabetes. NCDs have been the leading causes of death among women globally for at least the past three decades and are now responsible for two in every three deaths among women each year (1).

Several enduring myths have contributed to the neglect of NCDs in women (2). First, there is a strong and persistent view that only health-related issues of importance to women are defined through their reproductive capacity (3), yet two thirds of all deaths and disabilities in women relate to chronic diseases, violence, and other injuries. This myth in particular, reflects, in part, a gender bias (4). Second, NCDs, especially CVDs, have been considered primarily as diseases of men. Although age-specific NCD death rates in women lag behind the rates in men by about 10 years, the absolute number of NCD deaths in women (16.2 million) is similar to that of men (18.4 million) because women live longer – on average, between 6 and 8 years (5). The third myth is that NCDs in women are an issue only in high-income countries. In fact, most NCD deaths in women occur in low- and middle-income countries and the rates in these countries are much higher than in wealthy countries. The fourth and the most distressing myth is that NCDs cause deaths only in older people and ‘we have to die of something, so why bother with complicated conditions like NCDs’. Unfortunately, and especially now in low- and middle-income countries, NCD deaths are frequent in people under the age of 70 years, including women, and many of these deaths are slow and miserable. A large proportion of NCD deaths, especially before old age, are avoidable with cheap, cost-effective, and in some cases, cost-saving interventions.

The good news is that, despite the overall increasing number of NCD deaths in women due to increasing population size and aging, the age-specific death rates, especially for CVD, are declining, and in some countries, surprisingly quickly (6–8). In many countries, the decline began before governments or non-governmental organizations mounted awareness and prevention campaigns. It is likely that the initial decline in death rates was due to the diffusion of information from the early epidemiological studies, notably the Framingham Heart Study, and the powerful evidence on the relationship between tobacco use, lung cancer, and CVD that began to be disseminated in the 1960s.

More recently, treatments – especially drugs to manage high levels of blood pressure and cholesterol – have also played an important part in continuing the decline in death rates which now include lung cancer in men, but not yet women, as well as stomach, breast, and cervical cancers. The challenge now is to apply globally the interventions that have been so beneficial to women in high-income countries.

Risk factors for heart disease and stroke, the two leading causes of death among women, are similar for men and women. Factors such as age and family history play a role, but the majority of CVD deaths are due to modifiable risk factors such as tobacco use; diets high in fat, salt, and sugar; high blood pressure; high cholesterol; obesity; and diabetes. Because fewer women actively smoke – or have smoked for a shorter time – than men, and because they drink less and in less harmful ways than do men, in most parts of the world the major NCD risk factors cause less of a burden in women than in men (9) and therefore more ambitious risk factor targets will be required for women than for men to achieve the ‘25 by 25’ premature NCD mortality target set by WHO (10). Dietary risk factors have broadly similar effects for both women and men. With the exception of high body mass index and high fasting plasma glucose, most of the leading risk factors for NCDs have declined, especially in high-income countries (3). Obesity levels are a concern for women; almost everywhere, women are more obese than men. There are also significant differences in the way both symptomatic and asymptomatic women are treated compared to men. For example, gender, but not age, race, or social class of a patient significantly influenced doctors’ diagnostic and management activities in a study that controlled for these variables simultaneously. Women were asked fewer questions, received fewer examinations, and had fewer diagnostic tests ordered for coronary heart disease (11, 12). These differences are a reflection of the strong gender bias against equitable prevention and treatment of women (13).

The future of women and the treatment of NCDs is encouraging. There is an increasing recognition of the importance of a life course approach to the prevention of NCDs beginning with the health of girls and young women before and during pregnancy (14). The integration of NCD prevention activities into maternal and women-centric health programs has considerable potential since there is generally poor access to care for women, girls, and other vulnerable groups affected by NCDs (15). In many societies, women lack control over resources and, hence, cannot afford quality care for NCDs. Women also face sociocultural, geographic, and economic barriers to access to care. They are less recognized and catered to in terms of accessibility, comprehensiveness, and responsiveness of healthcare systems.

Furthermore, there have been major global initiatives to overcome the general neglect of NCDs. The UN High-Level Meeting on the Prevention and Control of NCDs in September 2011 was a turning point. Heads of state and governments made many major commitments to reverse the neglect of NCDs, and the health of women figures prominently in the political declaration from this meeting (16). In response to the UN meeting, member states of WHO have agreed upon a global goal to reduce avoidable NCD mortality by 25% by 2025 (‘25 by 25’), and a set of voluntary targets and indictors have also been agreed upon, although none of them are gender specific (17).

The targets agreed upon by WHO member states cover nine areas. The Lancet NCD Action Group has proposed a smaller set of priority targets – three to five – in the belief that it is important for countries, especially those beginning their prevention and management efforts, to concentrate on interventions which are among the ‘best buys’ in terms of health impact and will thus ensure rapid progress toward the ‘25 by 25’ goal (18). Of course, once experience and capacity builds, a broader range of interventions should be implemented. The Lancet NCD Action Group proposes a stepwise approach to NCD prevention beginning with sustained high-level leadership; multisectoral action; a focus on tobacco control and salt reduction; and the identification in primary health care of women and men at high overall risk of CVD (>30% over 10 years) and their treatment with cheap generic combination drugs (19).

Tobacco control is an excellent entry point for NCD prevention, and accelerated implementation of the Framework Convention on Tobacco Control is top priority. Tobacco use in high-income countries is at comparable levels among women and men, and in most of these countries, the prevalence of smoking is declining slowly as an increasing range of evidence-based cost-saving control measures are introduced. The most effective intervention is regular and substantial increases in the price of tobacco; plain packaging of tobacco products, first introduced in Australia in 2012, has opened a new phase in the fight against the tobacco industry.

Fortunately, in many low- and middle-income countries, for example, China and Indonesia, smoking rates are substantially lower in women than in men. This market represents a major opportunity for the tobacco industry, and it is doing its best to recruit new smokers, including enticing young women. Women use tobacco in a variety of forms, not just smoked tobacco. For this reason, the proposed WHO voluntary target is to reduce tobacco use by 30% by 2025. This target is not sufficiently ambitious and, following the lead of New Zealand which is committed to being essentially tobacco-free by 2025 (that is, a smoking prevalence of <5%), the Lancet NCD Action Group is proposing a tobacco-free world by 2040 (20). Secondhand smoke is a particular issue for women in low- and middle-income countries that do not yet have effective smoke-free environments (21). Effective legislation to protect people, especially women and children, from secondhand smoke is a priority.

Salt reduction will have an important effect on reducing population blood pressure levels through its impact on CVDs (22). Although the dominant approach in wealthy countries to lowering blood pressure is through medical treatment, this approach is not practical or affordable in most countries (23). Because about half the CVD events occur in people with ‘normal’ blood pressures, that is, below a systolic blood pressure of 140 mmHg, population salt reduction has the major advantage of leading to fewer CVD deaths in these people. Women will play a vital role in cultures where salt is overused; they can more easily take a leadership role at the household level in terms of reducing discretionary salt use. The agreed upon voluntary target is a 30% reduction in per capita salt intake by 2025; the original WHO target was a per capita intake of 5 g per day (24).

The priority treatment goal is to increase the coverage in primary health care of generic drugs for men and women at high risk of CVD. The dominant approach to the medical management of high risk is to treat individual elevated risk factors based on arbitrary cut points for ‘abnormality’. A much more efficient approach is to manage people at elevated overall risk based on their age, gender, and combined risk factor status; this approach will lead to a lower number of women on treatment – because of their lower overall risk – and better use of limited resources for the same or greater health impact. However, for this approach to reach its potential, it will need to be embraced by health professionals trained in the traditional medical model.

Assuring progress toward the NCD goal ‘25 by 25’, requires the establishment of an independent accountability mechanism, monitoring trends, reviewing progress, and acting as indicated to accelerate progress (25). A useful precedent has been set by the establishment of an independent expert advisory group to oversee progress toward the goals for Women's and Children's health; this model has relevance to NCDs. It is desirable for NCDs to be incorporated into the accountability mechanisms for other global health priorities.

As the 2015 deadline for the Millennium Development Goals approaches, discussions are underway on the post-2015 development agenda. It is critical that women and NCDs are central to the new human development agenda. So far, the prospects are promising with NCDs being recognized in the health consultations as being central to human development. If NCDs assume their rightful place post-2015, women throughout the world will benefit enormously.

## References

[1] Lozano R, Naghavi M, Foreman K, Lim S, Shibuya K, Aboyans V (2012). Global and regional mortality from 235 causes of death for 20 age groups in 1990 and 2010: a systematic analysis for the Global Burden of Disease Study 2010. Lancet.

[2] WHO (2005). Preventing chronic diseases: a vital investment.

[3] Bustreo F, Knaul FM, Bhadelia A, Beard J, de Carvalho IA (2012). Women's health beyond reproduction: meeting the challenges (editorial). Bull World Health Organ.

[4] Ruiz-Cantero MT, Vives-Cases C, Artazcoz L, Delgardo A, Garcia Calvente M, Miqueo C (2007). A framework to analyses gender bias in epidemiological research. J Epidemiol Community Health.

[5] Salomon JA, Wang H, Freeman MK, Vos T, Flaxman AD, Lopez AD (2012). Healthy life expectancy for 187 countries, 1990–2010: a systematic analysis of the Global Burden of Disease Study 2010. Lancet.

[6] Berg J, Bjork L, Lappas G, O'Flaherty M, Capewell S, Rosengren A (2014). Continuing decrease in coronary heart disease mortality in Sweden. BMC Cardiovasc Disord.

[7] Capewell S, O'Flaherty M (2011). Rapid mortality falls after risk factor changes in populations. Lancet.

[8] Hotchkiss JW, Davies CA, Dundas R, Hawkins N, Jhund PS, Scholes S (2014). Explaining trends in Scottish coronary heart disease mortality between 2000–2010 using IMPACTSEC model: retrospective analyses using routine data. BMJ.

[9] Lim SS, Vos T, Flaxman AD, Danaei G, Shibuya K, Adair-Rohani H (2013). A comparative risk assessment of burden of disease and injury attributable to 67 risk factors and risk factor clusters in 21 regions, 1990–2010: a systematic analysis for the Global Burden of Disease Study 2010. Lancet.

[10] Kontis V, Mathers CD, Rehm J, Stevens GA, Shield KD, Bonita R (2014). Contribution of six risk factors to achieving the 25×25 non-communicable disease mortality reduction target: a modelling study. Lancet.

[11] Chang AM, Mumma B, Keara L, Sease K, Robey JL, Shofer FS (2007). Gender Bias in Cardiovascular Testing Persists after Adjustment for Presenting Characteristics and Cardiac Risk. Academic Emergency Medicine.

[12] Arber S, McKinlay J, Adams A, Marceau L, Link C, O'Donnel A (2006). Patient characteristics and inequalities in doctors’ diagnostic and management strategies relating to coronary heart disease: a video-simulation experiment. Soc Sci Med.

[13] Risberg G, Johansson EE, Hamberg K (2009). A theoretical model for analyzing gender bias in medicine. Int J Equity Health.

[14] Power C, Kuh D, Morton S (2013). From developmental origins of adult diseases to life course research on adult disease and ageing: insights from birth cohort studies. Annu Rev Public Health.

[15] Maina WK (2011). Integrated non-communicable disease prevention into maternal and child health programs: can it be done and what will it take?. Int J Gynaecol Obstet.

[16] United Nations Political declaration of the high-level meeting of the general assembly on the prevention and control of non-communicable diseases. http://www.who.int/nmh/events/un_ncd/political_declaration_/en.pdf.

[17] World Health Organization NCD global monitoring framework: ensuring progress on non-communicable diseases in countries. http://www.who.int/nmh/global_monitoring_framework/en.

[18] Bonita R, Magnusson R, Bovet P, Zhao D, Malta DC, Geneau R (2013). Country actions to meet the UN commitments on non-communicable diseases: a stepwise approach. Lancet.

[19] Lim SS, Gaziano TA, Gakidou E, Reddy KS, Farzadfar F, Lozano R (2007). Prevention of cardiovascular disease in high-risk individuals in low-income and middle-income countries: health effects and costs. Lancet.

[20] Beaglehole R, Bonita R, Horton R, Ezzati M, Bhala N, Amuyunzu-Nyamongo M (2012). Measuring progress on NCDs: one goal and five targets. Lancet.

[21] Oberg M, Jaakkola MS, Woodward A, Peruga A, Pruss-Ustun A (2011). Worldwide burden of disease from exposure to second-hand smoke: a retrospective analysis of 193 countries. Lancet.

[22] He FJ, Brinsden HC, MacGregor GA (2013). Salt reduction lowers cardiovascular risk: meta-analysis of outcome trials. Lancet.

[23] Tang JL, Hu YH (2005). Drugs for preventing cardiovascular disease in China. Br Med J.

[24] World Health Organization (2012). Sodium for adults and children: guidelines. http://www.who.int/nutrition/publications/guidelines/sodium-intake/en.

[25] Beaglehole R, Bonita R, Horton R (2013). Independent accountability for NCDs. Lancet.

